# TRF2 and lamin A/C interact to facilitate the functional organization of chromosome ends

**DOI:** 10.1038/ncomms6467

**Published:** 2014-11-17

**Authors:** Ashley M. Wood, Jannie M. Rendtlew Danielsen, Catherine A. Lucas, Ellen L. Rice, David Scalzo, Takeshi Shimi, Robert D. Goldman, Erica D. Smith, Michelle M. Le Beau, Steven T. Kosak

**Affiliations:** 1 Department of Cell and Molecular Biology, Feinberg School of Medicine, Northwestern University, Chicago, Illinois 60611, USA; 2 Division of Basic Sciences, Fred Hutchinson Cancer Research Center, Seattle, Washington 98109, USA; 3 Section of Hematology/Oncology, Department of Medicine and Comprehensive Cancer Center, The University of Chicago, Chicago, Illinois 60637, USA

## Abstract

Telomeres protect the ends of linear genomes, and the gradual loss of telomeres is associated with cellular ageing. Telomere protection involves the insertion of the 3′ overhang facilitated by telomere repeat-binding factor 2 (TRF2) into telomeric DNA, forming t-loops. We present evidence suggesting that t-loops can also form at interstitial telomeric sequences in a TRF2-dependent manner, forming an interstitial t-loop (ITL). We demonstrate that TRF2 association with interstitial telomeric sequences is stabilized by co-localization with A-type lamins (lamin A/C). We also find that lamin A/C interacts with TRF2 and that reduction in levels of lamin A/C or mutations in *LMNA* that cause an autosomal dominant premature ageing disorder—Hutchinson Gilford Progeria Syndrome (HGPS)—lead to reduced ITL formation and telomere loss. We propose that cellular and organismal ageing are intertwined through the effects of the interaction between TRF2 and lamin A/C on chromosome structure.

## Abstract (web summary)

The shortening of telomeres—a structure that protects chromosome ends—is associated with cellular aging. Here, Wood *et al.* present evidence that interaction between the telomere-binding protein TRF2 and lamin A/C facilitates the formation of interstitial t-loops and stabilizes telomeres.

## Graphical abstract

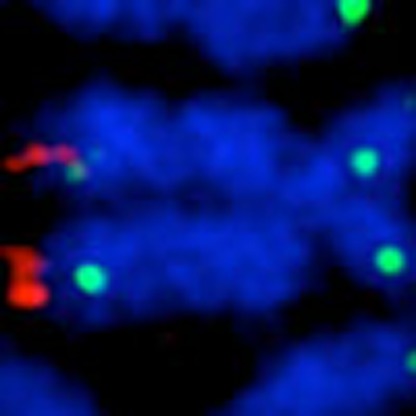

Regulation and maintenance of eukaryotic chromosome structure is necessary to ensure proper gene regulation, DNA replication and cell division. Chromosome ends are comprised of telomeric repeats and represent unique challenges in chromosome structure. First, there is a mechanistic problem associated with the replication of telomere ends, which can be overcome by expression of telomerase. However, telomerase expression is usually lost in human cells during cell differentiation, and telomere shortening is a fundamental aspect of cellular ageing or replicative senescence. Second, telomeres must be protected against recognition by DNA damage machinery. The shelterin complex is one way that cells manage this second challenge.

In vertebrates, telomere repeat-binding factors (TRFs) 1 and 2 (also known as TERFs) are core members of the shelterin complex that bind duplex telomeric DNA as homodimers^1^,^2^,^3^. TRF1 is a negative regulator of telomere length and is suggested to induce bending, looping and pairing of telomeric DNA^4^,^5^,^6^. Although structurally similar to TRF1, TRF2 is functionally divergent as it facilitates strand invasion of 3′ single-stranded overhangs found at chromosome ends into duplex telomeric DNA, forming structures called t-loops^7^,^8^,^9^,^10^,^11^,^12^. TRF2 has also been shown to bind at internal genomic sites, mostly at 5′-TTAGGG-3′ repeat sequences referred to as interstitial telomeric sequences (ITSs)^13^,^14^. Loss of TRF2 function leads to increased activity of the DNA damage response pathway, end-to-end chromosome fusion and cellular senescence^1^,^15^,^16^,^17^,^18^. Here we provide evidence indicating that TRF2-dependent t-loops can also form at ITSs, forming a structure that we term an interstitial t-loop (ITL), and suggest that this novel chromosome-end structure is facilitated by an interaction between TRF2 and the nuclear intermediate filament protein lamin A/C. Specific mutations in the *LMNA* gene are associated with the premature ageing disorder Hutchinson Gilford Progeria Syndrome (HGPS), the most common of which results in activation of a cryptic splice site that leads to a shortened, permanently farnesylated form of lamin A known as progerin^19^,^20^. We provide evidence that a reduction in levels of TRF2 or lamin A/C, dominant negative TRF2 expression and the *LMNA* mutation causing HGPS lead to reduced ITL and a striking degree of telomere loss. We propose that cellular and organismal ageing are intertwined through the effects of the interaction between TRF2 and lamin A/C on chromosome structure.

## Results

### Chromosome ends are inverted in metaphase chromosomes

**Figure 1:**
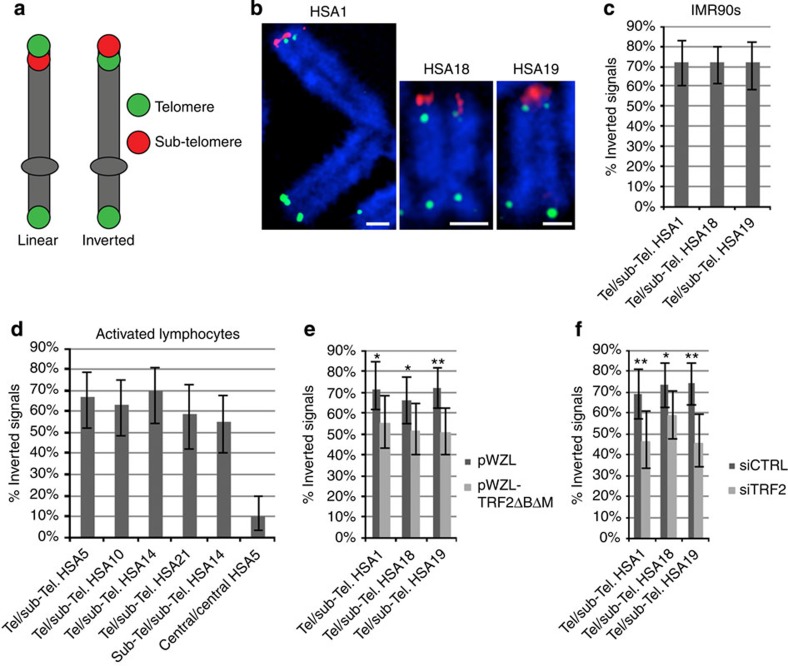
Chromosome ends show an inverted orientation. (**a**) Schematic of linear versus inverted chromosome structure. (**b**) Super-resolution images of IMR90 chromosomes HSA1, HSA18 and HSA19 with inverted telomere/subtelomere chromosome structure. A telomere FISH probe is in green, and a subtelomeric FISH probe specific to each chromosome is in red. DNA is stained with DAPI. Scale bar, 1 μm. FISH signal orientation for the indicated probes was quantified for (**c**) IMR90s (*n*=54–89 signals per probe set) and (**d**) activated lymphocytes (*n*=37–50 signals per probe set). The frequency of inverted FISH signal orientation was also quantified for IMR90s (**e**) overexpressing pWZL or pWZL-TRF2ΔBΔM (*n*=56–87 signals per probe set) and (**f**) cells treated with siCTRL or siTRF2 (*n*=55–70 signals per probe set). Error bars represent 95% confidence intervals for all quantifications. *P<0.05, **P<0.01, Student’s *t*-test.

Examination of any telomere fluorescence *in situ* hybridization (FISH) study on mitotic chromosomes reveals an interesting phenomenon: telomeric FISH signal is often found more internal than the most distal position on the chromosome relative to the centromere, as the linear organization of the genome necessitates^21^,^22^,^23^ (Supplementary Fig. 1a). To further interrogate this unexpected observation, we designed FISH probes that map near the ends of chromosomes but centromeric to telomeric repeat regions, here referred to as subtelomeric probes (Supplementary Fig. 2). Probes were chosen for HSA1, HSA18 and HSA19, representing a large chromosome, a small gene-poor chromosome and a small gene-rich chromosome, respectively. On the basis of the organization of the linear genome, it is expected that two-colour FISH using these subtelomeric probes and a telomeric probe would show the telomeric probes at the chromosome end (linear; Fig. 1a). However, we observed a significant number of inverted signals in metaphase spreads from primary human lung fibroblasts (IMR90s), such that the telomeric probe is more centromeric than subtelomeric probes (inverted; Fig. 1a–c).

We extended our analysis to activated human lymphocytes and to include probes on the p and q arms of chromosomes. We again observed a significant frequency of inverted chromosome ends for all chromosomes tested, highlighting the prevalence of this phenomenon across chromosomes and cell-types (Fig. 1d; Supplementary Fig. 1e). To examine the extent of this inversion along the chromosome, subtelomeric chromatin regions were compared with two probes recognizing regions of HSA14 located within 1 Mb of the telomere (Supplementary Fig. 2). Two-colour FISH in activated lymphocytes revealed an inverted signal in over half of the chromosomes analysed (Fig. 1d). Probes on HSA5 located 4 Mb from the telomere (central HSA5) were also analysed (Supplementary Fig. 2), and show that these more central sequences almost always localize in the linear orientation, indicating that the inverted structure is specific to chromosome ends (Fig. 1d). All together, an inverted chromosome-end structure is observed for seven different chromosomes and two different primary cell types with no obvious genomic instability. Furthermore, this inverted chromosome structure was also observed in mouse cells, indicating that the phenomenon is conserved across species (Supplementary Fig. 1f). We suggest that the observed inversion at mitotic chromosome ends is a widespread feature of vertebrate chromosomes and is the result of chromosome looping.

As mentioned previously, chromosome looping within telomeres (t-loops) is dependent on TRF2. To determine whether the inverted chromosome-end structure involving non-telomeric DNA that we observe requires TRF2, we performed FISH analysis on mitotic chromosomes in IMR90s subjected to either TRF2 siRNA (siTRF2) knockdown or expression of a dominant negative TRF2 (pWZL-TRF2ΔBΔM). This dominant-negative TRF2 allele lacks the amino-terminal basic domain and carboxy-terminal DNA-binding domain, and expression of this construct results in the removal of endogenous TRF2 from telomeres^24^. Compared with control knockdown cells (siCTRL) or control overexpression cells (pWZL), we observed a significant reduction in the frequency of inverted chromosome ends for all three of the chromosomes tested when TRF2 is disrupted (Fig. 1e,f; Supplementary Fig. 1d). This suggests that like t-loop formation, the inverted chromosome-end structure is TRF2 dependent. As mentioned previously, TRF2 disruption leads to telomere instability and cell senescence^1^,^15^,^16^,^17^,^18^. For the analysis shown here, we chose early time points after TRF2 disruption, so that cells were still undergoing cell division and telomere FISH signal was still present in the majority of chromosomes. Therefore, the data presented likely underrepresent the detrimental effect that TRF2 disruption has on this inverted chromosome-end structure.

### Evidence for chromosome-end looping in interphase

**Figure 2:**
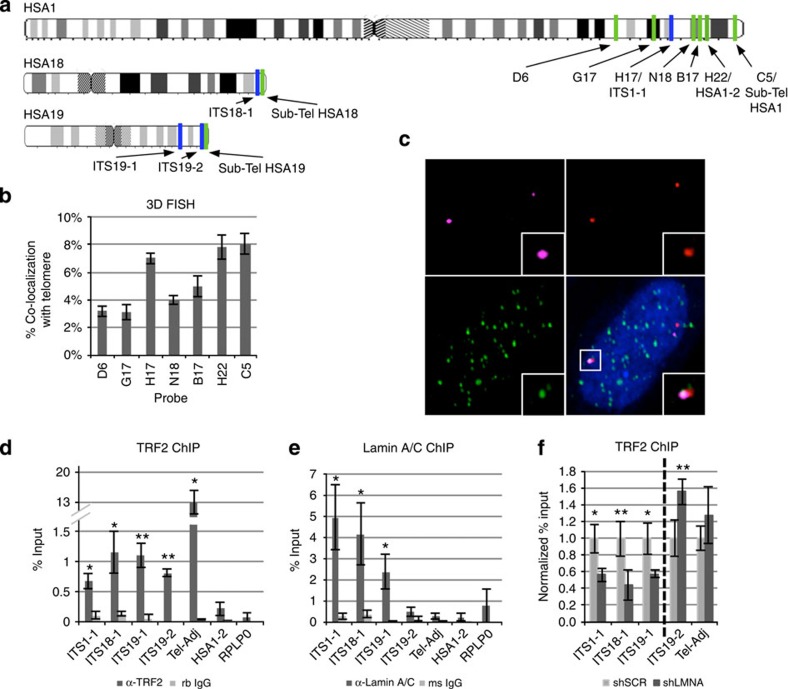
TRF2 and lamin A/C are enriched at ITSs. (**a**) The localization of BACs used for mitotic and interphase FISH analysis and primers used for ChIP–qPCR analysis are indicated. (**b**) Quantification of the co-localization between BACs on HSA1 and telomere signal in IMR90 interphase nuclei. (**c**) Representative image of 3D FISH analysis quantified in **b** showing the H17 BAC (magenta), the H22 BAC (red), telomere FISH signal (green) and DNA stained with DAPI (blue). One of the alleles (white box) is enlarged in the bottom right depicting co-localization of H17, but not H22, with the telomere. (**d**,**e**) TRF2 and lamin A/C ChIP–qPCR analysis at four ITSs (ITS1-1, ITS18-1, ITS19-1 and ITS19-2), a sequence immediately adjacent to the telomere (Tel-Adj), and sites without an ITS (HSA1-2 and RPLP0). (**f**) TRF2 ChIP–qPCR analysis at sites identified as positive in **d**, performed with cells treated with shLMNA or shSCR. Sites to the left of the dotted line show lamin A/C association in **e**, and sites to the right of the dotted line do not. Values are normalized to those of shSCR and data are presented as mean±s.e.m. (*n*=3). *P<0.05, **P<0.01, Student’s *t*-test.

To extend the analysis of chromosome-end structure to interphase nuclei, we analysed regions along the q arm of HSA1 for co-localization with telomeres by FISH in IMR90s (Fig. 2a–c; Supplementary Fig. 3a). As expected^25^, we saw a decrease in the frequency of co-localization of HSA1 probes and telomere signal as the probe is linearly located further from the chromosome end. However, one probe in particular, H17, shows more frequent co-localization with telomere signal than expected based on its distance from the chromosome end (Fig. 2b; Supplementary Fig. 3b). We predict that this increase in co-localization frequency is due to a chromosome loop between this region of HSA1 and the telomere in interphase nuclei. To demonstrate that the telomere physically interacts with the H17 region of HSA1, we performed a telomere pull-down assay. This assay involves crosslinking chromatin followed by telomere pull down using a biotinylated telomere-specific probe to precipitate telomeric DNA as well as other DNA (or protein) within crosslinking distance of the telomere^26^. Using this assay, we confirmed that the H17 region is highly enriched for interaction with the telomere over surrounding regions of HSA1 (Supplementary Fig. 3c).

We hypothesize that the chromosome organization revealed in our FISH and telomere pull-down analyses represents formation of ITLs, t-loops formed by telomere association with the abundant ITSs found throughout the human genome. On the basis of the sequence requirements identified by biochemical characterization of TRF2 binding to DNA^27^,^28^,^29^,^30^ and genome-wide TRF2 studies^13^,^14^, we define ITSs as sites that contain a dimeric telomeric repeat (5′-TTAGGGTTAGGG-3′) or at least two nine-nucleotide telomeric tracts (5′-TTAGGGTTA-3′) within 100 bp (Supplementary Fig. 4a). On the basis of these criteria, we identified two ITSs within the H17 bacterial artificial chromosome (BAC) (here collectively referred to as ITS1-1), but found that the other HSA1 BACs are devoid of ITSs. We therefore tested for TRF2 association with ITS1-1 by chromatin immunoprecipitation (ChIP) and quantitative PCR (qPCR) and found that TRF2 does in fact associate with this region (Fig. 2d). Furthermore, we identified ITSs on HSA18 and HSA19 (ITS18-1, ITS19-1 and ITS19-2) positioned just centromeric to the probes used in Fig. 1c and also observed enriched association of TRF2 at these loci by ChIP (Fig. 2a,d). This analysis shows that TRF2 binding is enriched at ITSs on HSA1, HSA18 and HSA19 that are positioned to induce the proposed ITL.

TRF2 association with a telomere-adjacent sequence (Tel-Adj) is notably higher than TRF2 binding at ITSs (Fig. 2d). This difference is likely due to the enrichment of telomeric repeats in the Tel-Adj region relative to ITSs. Furthermore, genome-wide studies have shown that TRF2 associates with some ITSs, but not all^13^,^14^, and it has therefore been difficult to predict *in vivo* TRF2 binding based on DNA sequence alone. Together, these findings suggest that interstitial TRF2 binding and the proposed ITL may require additional DNA-associated proteins.

### TRF2 binding at ITSs is stabilized by lamin A/C

Lamins are highly conserved intermediate filament proteins that make up the nuclear lamina and are localized throughout the nucleoplasm^31^,^32^,^33^. There is growing evidence in the literature that suggests a link between telomeres and the nuclear lamina, which includes a role for lamin A/C in regulating telomere length and positioning^34^,^35^,^36^,^37^,^38^,^39^,^40^. Therefore, lamin A/C is a likely candidate for playing a role in the putative ITL.

We identified 345 ITSs that overlap with lamin A/C genome-wide ChIP^41^ and/or DamID (DNA adenine methyltransferase identification)^42^ data sets. In addition, we found that TRF2-binding sites previously identified by ChIP^13^,^14^ are closer to lamin A/C sites than expected for a random distribution (Supplementary Fig. 4b). To determine whether lamin A/C associates with ITSs, we performed ChIP–qPCR analysis in IMR90s and find lamin A/C enrichment at three of the four TRF2-associated ITSs identified in Fig. 2d (Fig. 2e). We then tested whether lamin A/C affects TRF2 binding at these ITSs using lamin A/C knock down with LMNA shRNA (shLMNA) or a scrambled version of the construct (shSCR; Supplementary Fig. 5a,b). We find that a reduction in lamin A/C leads to a significant decrease in TRF2 binding at the three ITSs that associate with both lamin A/C and TRF2 (Fig. 2f). This effect is specific to lamin A/C-associated sites since no reduction in TRF2 association is observed at either the fourth ITS that does not associate with lamin A/C (ITS19-2) or at the telomere-adjacent site (Tel-Adj; Fig. 2f). These data indicate that a subset of ITSs is bound by both TRF2 and lamin A/C and that lamin A/C stabilizes TRF2 binding at these sites.

### TRF2 interacts with lamin A/C

**Figure 3:**
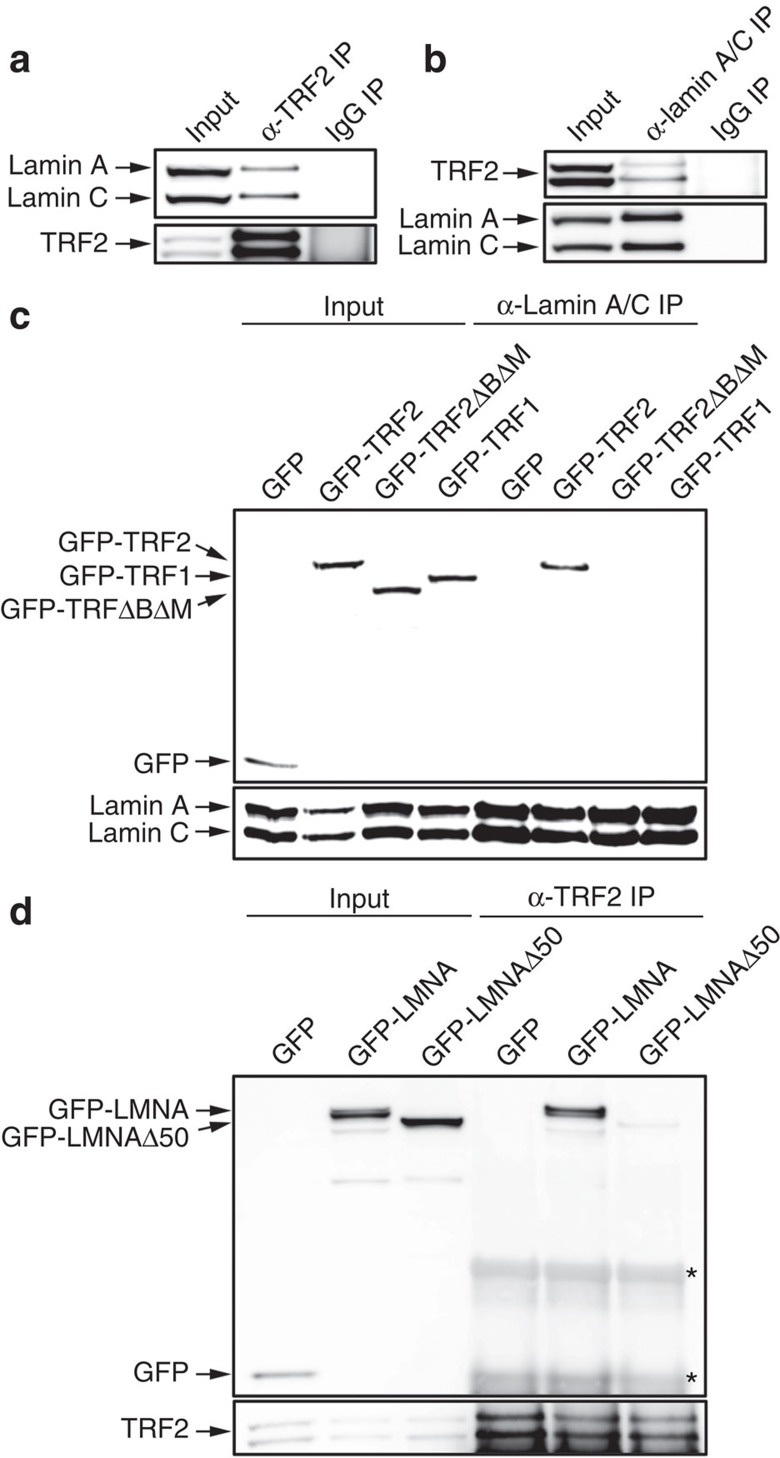
TRF2 interacts with lamin A/C. (**a**) Pull down of endogenous TRF2 in IMR90s. The top panel shows a blot probed for endogenous lamin A/C, and the bottom panel shows a blot with the same samples probed for TRF2. (**b**) Pull down of endogenously expressed lamin A/C in IMR90s. The top panel shows a blot probed for endogenous TRF2, and the bottom panel shows a blot with the same samples probed for endogenous lamin A/C. (**c**) Pull down of endogenous lamin A/C in IMR90s exogenously expressing GFP, GFP-TRF2, GFP-TRFΔBΔM or GFP-TRF1. The top panel shows a blot probed for GFP, and the bottom panel shows the same blot probed for lamin A/C. (**d**) Pull down of endogenous TRF2 in IMR90s exogenously expressing GFP, GFP-LMNA or progerin (GFP-LMNAΔ50). The top panel shows a blot probed for GFP, and the bottom panel shows the same blot probed for TRF2. Asterisks denote IgG bands.

The functional interaction between lamin A/C and TRF2 at ITSs suggests a possible molecular interaction between these two proteins. To test for this interaction, we performed co-immunoprecipitation analysis with endogenous TRF2 and lamin A/C. Immunoprecipitation of TRF2 revealed pull down of lamin A/C as well as the obverse (Fig. 3a,b; Supplementary Fig. 6a–d). To determine the specificity of this interaction, we examined the interaction between endogenous lamin A/C and wild-type GFP-TRF2, GFP-TRF2ΔBΔM^24^ or wild-type GFP-TRF1 expressed in IMR90s. This analysis further confirmed the interaction with TRF2, but did not show an interaction between lamin A/C and TRF1, suggesting that the interaction is specific to TRF2 (Fig. 3c; Supplementary Fig. 6e,f). Furthermore, the lack of interaction with TRF2ΔBΔM, a mutant allele lacking the DNA-binding domain of TRF2, suggests that lamin A/C only interacts with functional, DNA-bound TRF2.

The *LMNA* mutation in HGPS patients results in activation of a cryptic splice site that leads to a shortened, permanently farnesylated form of lamin A known as progerin^19^,^20^,^43^. At the organismal level, this mutation leads to symptoms of early ageing such as scleroderma skin conditions, atherosclerosis, kidney failure, loss of eyesight, and cardiovascular problems^20^. The cellular phenotypes that result include defective nuclear morphology, loss of heterochromatin, premature cellular senescence, chromosomal segregation defects, chronic DNA damage response and telomere shortening^34^,^44^,^45^,^46^,^47^,^48^,^49^. Since progerin expression closely mimics the deleterious effects of reduced levels of lamin A/C on telomeres, we predict that progerin is unable to interact properly with TRF2. To test this hypothesis, we performed co-immunoprecipitation analysis by immunoprecipitating endogenous TRF2 and probing for GFP-lamin A/C or GFP-progerin (GFP-LMNAΔ50; Fig. 3d; Supplementary Fig. 6g,h). In agreement with the endogenous pull-down assay, GFP-lamin A/C precipitates with TRF2; however, GFP-progerin does not, suggesting that only wild-type lamin A/C stably interacts with TRF2.

### Lamin A/C is necessary for chromosome-end organization

**Figure 4:**
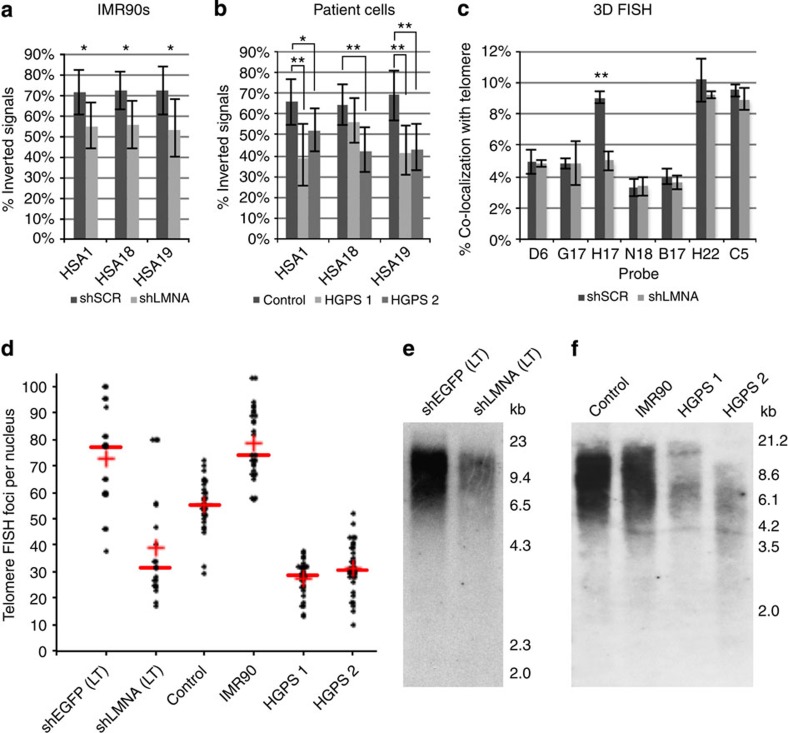
Lamin A/C stabilizes chromosome-end structure and is necessary for telomere stability. The frequency of inverted chromosome-end structure was quantified for HSA1, HSA18 and HSA19. For each chromosome, the positioning of a telomeric probe signal was compared with the positioning of the indicated subtelomeric probe signal. This analysis was performed for (**a**) IMR90s treated with shLMNA or shSCR (*n*=45–89 signals per probe set) *P<0.05, Student s t-test and (**b**) fibroblasts from a healthy patient (control) and two different HGPS patients (HGPS 1 and HGPS 2; *n*=39–83 signals per probe set). *P<0.05, **P<0.01, Student’s *t*-test. (**c**) Quantification of IMR90 interphase nuclei treated with shLMNA or shSCR for co-localization between BACs on HSA1 (see Fig. 2a) and telomere signal. Results are mean±s.e.m. (*n*=3). **P<0.01, Student’s *t*-test. (**d**) Quantification of the number of telomeric FISH foci per nucleus in shLMNA(LT) or shEGFP(LT) IMR90s, as well as control or HGPS patient cells. The red line and cross indicate the data set median and mean, respectively. (**e**) Southern blot analysis of telomeric DNA size and abundance in shLMNA(LT) or shEGFP(LT) IMR90s. (**f**) Southern blot analysis of telomeres from control patient cells and HGPS patient cells, telomeric DNA from IMR90s is included for comparison.

We propose that the interaction between lamin A/C and TRF2 stabilizes telomeres by facilitating ITL formation. Since lamin A/C knockdown results in a reduction in TRF2 binding to ITSs, our model predicts that lamin A/C knockdown will also decrease the frequency of inverted chromosome ends. To test this, we again treated IMR90s with shLMNA or shSCR and found that the frequency of inverted chromosome ends was significantly reduced in the lamin A/C knockdown for all three of the chromosomes tested (Fig. 4a). Furthermore, since we have shown that TRF2 does not interact with progerin, we performed this analysis in cells from HGPS patients with the canonical *LMNA* mutation (G608G) and in control cells from a healthy individual. In these cells, we saw a significant reduction in inverted chromosome ends for all three chromosomes in one HGPS patient and in two out of three chromosomes in the second HGPS patient (Fig. 4b). We also observed a reduction in H17 co-localization with telomere signal in interphase nuclei treated with shLMNA, but no change in the other BACs analysed on HSA1 (Fig. 4c; Supplementary Fig. 7). Furthermore, we showed that this decrease in H17 co-localization with the telomere is not due to changes in nuclear volume during shLMNA knockdown (Supplementary Fig. 5c). Together, these data are consistent with the hypothesis that lamin A/C is necessary for ITL in both mitosis and interphase. As evidenced below, telomere FISH signal is dramatically lost in the absence of functional lamin A/C. Since we can only perform these experiments on chromosomes and nuclei that maintain detectable telomere FISH signals, we are by necessity analysing chromosomes that have not yet experienced gross telomere defects due to the loss of lamin A/C. Therefore, our data likely underestimate the actual effect of loss of lamin A/C function on chromosome-end organization.

### Lamin A/C is necessary for telomere protection

If ITL is indeed a mechanism of telomere protection, disruption of inverted chromosome ends should lead to telomere instability. Increased telomere shortening has been reported in fibroblasts isolated from HGPS patients as well as in mouse embryonic fibroblasts derived from *LMNA*-deficient mice^34^,^35^. Furthermore, telomere defects (including loss of telomere signal when detected by FISH) are observed in fibroblasts overexpressing progerin and in *LMNA*-deficient mouse embryonic fibroblasts^35^,^50^. However, the effect of reduced lamin A/C expression on telomeres in human cells is not well characterized. Therefore, we analysed the telomeres of IMR90 fibroblasts selected for long-term expression of *LMNA* shRNA (shLMNA(LT)) or a control (shEGFP(LT)), as well as HGPS and control patient cells by FISH. These experiments revealed an overall reduction in the number of telomere foci per nucleus in fibroblasts expressing shLMNA(LT) compared with shEGFP(LT) (Fig. 4d; Supplementary Fig. 8a) and in HGPS patient cells compared with control cells (Fig. 4d). However, loss of signal in telomere FISH can be attributed to loss of telomeres or extremely shortened telomeres that fall below the threshold of detection. To distinguish between these two possibilities, we visualized telomeres by Southern blot analysis, revealing that the reduction in telomeric signal by FISH was due to an actual genomic loss of telomeres, observed as a decrease in signal in addition to telomere shortening, observed as a shift in size (Fig. 4e,f). The loss of telomeric DNA was more pronounced than telomere shortening. Moreover, this telomere loss was not a result of general genome instability in that the comparative genomic hybridization (CGH) revealed no gross genomic abnormalities after treatment with shLMNA(LT) (Supplementary Fig. 8b). Although many factors could contribute to the loss of telomere signals in shLMNA(LT) and HGPS cells, these data suggest that disruption of inverted chromosome ends may be a critical factor contributing to this phenotype.

## Discussion

Our data describe a novel form of mammalian chromosome organization that involves the interaction of telomeres with ITSs and nuclear lamins. Previous studies investigating t-loop structures in mammalian cells did not observe the ITL described here, likely because these studies specifically removed non-telomeric DNA from analysis^7^,^12^ or specifically visualized only telomeric DNA^10^. The methodology used here allows for analysis of the entire chromosome and therefore identified this novel chromosome-end structure.

A relationship between telomere disruption and HGPS has been previously suggested; however, the findings presented here provide a molecular link that involves an interaction between TRF2, a telomere-binding protein, and lamin A/C, the key protein disrupted in HGPS. We show that progerin, the mutant form of lamin A/C produced in HGPS cells, does not interact with TRF2 (Fig. 3d) and propose that this disrupted interaction leads to the telomere instability observed in HGPS. Interestingly, the 50 amino acids of lamin A/C deleted to produce progerin are present in lamin A but not in lamin C, although both lamin A and lamin C interact with TRF2 (Fig. 3a). This suggests that it is not necessarily the deletion, but more likely the improper processing of progerin that abrogates its interaction with TRF2. Moreover, we propose that the interaction between TRF2, lamin A/C and ITSs in normal cells is a regulated process. Our co-immunoprecipitation assays illustrate that only a small fraction of the total TRF2 and lamin A/C interacts. These results are expected due to the multitude of roles and interacting partners that each of these proteins has in the nucleus, and understanding the mechanisms that dictate when these proteins interact will provide interesting insight into how this complex is regulated.

Furthermore, the finding that not all TRF2-bound ITSs associate with lamin A/C suggests that ITSs are not all equivalent and raises the intriguing possibility that these interactions may be dynamic. The binding of TRF2 and lamin A/C to ITS may vary between cell types and/or during changes in chromatin state, and may influence the degree of chromatin looping and compaction of chromosomes at a given point in time.

Due to the techniques used in our analysis, we have specifically assayed the frequency of inverted chromosome ends in chromosomes that have detectable telomere signal and therefore have not yet experienced massive telomere loss. We therefore suggest that reversal of ITL by disruption of TRF2 or lamin A/C leads to exposed chromosome ends that are susceptible to recognition by DNA damage machinery, and we propose that reduced ITL precedes telomere instability. However, it is also possible that disruption of TRF2 or lamin A/C first leads to DNA damage at telomeres^18^,^35^,^47^,^50^, which then results in the observed reduction in inverted chromosome ends. Future analysis will distinguish between these two scenarios and will continue to increase our understanding of the interaction between lamin A/C and telomere stability. Nevertheless, ITL is a previously unidentified chromosome-end structure that we suggest is characteristic of stable, intact telomeres.

HGPS results in decreased lifespan^51^ and progerin accumulation has also been detected in elderly individuals with wild-type *LMNA*, supporting the idea that HGPS models normal human ageing^52^,^53^,^54^. We propose that lamin A/C- and TRF2-mediated ITL is necessary to promote a protective telomeric state, and that disruption of this state leads to cellular ageing through telomere loss, as well as accelerated organismal ageing. Moreover, the results we describe may also have a bearing on various structural defects that involve telomeres and disease, including translocations and alternative lengthening of telomeres (an important means for cellular transformation). The link between cellular and organismal ageing evidenced in the functional interaction we characterize here lends itself to a re-evaluation of the importance of the structure of chromosome ends in development and disease.

## Methods

### Cell culture

Normal human primary lung fibroblasts (IMR90s) were obtained from American Type Culture Collection (ATCC) and IMR90s selected for long-term expression of lamin A/C shRNA (shLMNA(LT)) or an EGFP (enhanced green fluorescent protein) control shRNA (shGFP(LT)) were kindly provided by Brian Kennedy (University of Washington, Seattle) and generated as previously described^55^. Primary dermal fibroblast cell lines from a healthy donor (AG13334) and HGPS patients (Patient 1: AG11498 and Patient 2: AG01972) were obtained from Coriell Cell Repository. IMR90s and patient cells were grown in Minimum Essential Medium alpha (Gibco, 12561-056) supplemented with 15% fetal bovine serum, 2 mM L-glutamine, 100 U ml^−1^ penicillin and 100 μg ml^−1^ streptomycin at 37 °C in 5% CO_2_. Mitogen-stimulated peripheral blood lymphocytes (activated lymphocytes) were obtained from healthy individuals as previously described^56^.

Lamin A/C or control shRNA (short hairpin RNA) knockdown and pWZL or pWZL-TRF2ΔBΔM overexpression were performed by retroviral transduction. GP-293 packaging cells (Clontech) cultured in DMEM supplemented with 10% fetal bovine serum, 2 mM L-glutamine, 100 U ml^−1^ penicillin and 100 μg ml^−1^ streptomycin were transfected with polyJET reagent (SignaGen Laboratories). In brief, 5 ml of fresh media was added to cells in a 10-cm dish. Then, 14.29 μg of expression vector and 0.71 μg VSV-G vector were diluted in 500 μl serum-free DMEM, and 45 μl of polyJET was diluted in 500 μl serum-free DMEM. Diluted polyJET solution was added to diluted DNA and incubated 15 min at room temperature. PolyJET/DNA solution was added dropwise to GP-293 cells and the cells were incubated for about 16 h at which point the media were changed. After 24 additional hours of incubation, the supernatant was collected, filtered, diluted 1:6 in MEM-alpha with 4 μg ml^−1^ polybrene and applied to IMR90 cells. A second infection was performed after an additional 24 h in the same manner. Selection was carried out on infected cells for 3 days with 3 μg ml^−1^ puromycin or 4 days with 200 μg ml^−1^ hygromycin. Post selection, cells were allowed to recover in normal media for 6 days for shRNA knockdown or 5 days for TRF2ΔBΔM overexpression before harvesting.

For siRNA knockdown, either TRF2 siRNA (sc-38505, Santa Cruz) or control siRNA (sc-36869, Santa Cruz) were introduced to IMR90 cells with PepMute siRNA Transfection Reagent (SignaGen Laboratories). In brief, siRNAs were diluted to 5 nM in Transfection Buffer. For a 10-cm plate, 20 μl of PepMute Reagent was added, and after incubation for 15 min at room temperature, the transfection mix was added to cells. Cells were harvested after 72 h.

### DNA constructs

EGFP-TRF constructs were generated by digesting the TRF1, TRF2 or TRF2ΔBΔM cDNAs out of the p16-1 vector, kindly provided by Titia de Lange (Rockefeller University, New York) and subcloning them into the pEGFP vectors (Clontech), resulting in EGFP fused to the N terminus of the TRFs. A pEGFP-NLS construct in the Clontech backbone was used as a control. GFP-lamin A/C and GFP-progerin constructs were kindly provided by David A. Jans (Monash University, Melbourne). pWZL and pWZL-TRF2ΔBΔM^24^ (Addgene plasmid 18013) were constructed by Titia de Lange (Rockefeller University, New York). The shLMNA and shSCR constructs^57^ have the target sequences: *LMNA* T1, 5′-AGCAGTCTCTGTCCTTCGA-3′ (human *LMNA*) and Neg-ctrl, 5′-ATGTACTGCGCGTGGAGA-3′ (scrambled).

### Coimmunoprecipitations

Adherent cells (2 × 10^7^) were washed with phosphate-buffered saline (PBS) and lysed in nondenaturing lysis buffer (1% Triton X-100, 50 mM Tris, pH 7.4, 300 mM NaCl, 5 mM EDTA, 0.02% sodium azide+protease inhibitors (Roche Complete Protease Inhibitor Cocktail)) for 40 min on ice. Lysates were scraped from plates, and insoluble material was pelleted. The lysate was then rocked for 2 h at 4 °C with 3 μg of the indicated antibody and then 30 μl of protein G Dynabeads were added and the mixture was rocked an additional hour at 4 °C. Following incubation, beads were washed three times with wash buffer (0.01% Tween 20/PBS), and beads were resuspended in 2 × SDS loading buffer (Invitrogen) with reducing agents and eluted sample was run on a 4–12% SDS–PAGE gel. Gel was transferred to a nitrocellulose membrane and probed for 2 h at room temperature or overnight at 4 °C with primary antibody followed by an appropriate secondary antibody conjugated to horseradish peroxidase (Abcam) for 1 h at room temperature. Membrane was washed with TBST (0.05% Tween 20/Tris-buffered saline) and detected with ECL Plus Western Blotting Detection Reagents (Amersham). Antibodies for IP: polyclonal goat α-lamin A/C (sc-6215, Santa Cruz) and rabbit α-TRF2 (NB110-57130, Novus Biologicals). Antibodies for western blot: 1:2,500 monoclonal mouse α-GFP (ab1218, Abcam), 1:2,000 rabbit α-TRF2 (NB110-57130, Novus Biologicals) and 1:5,000 monoclonal mouse α-lamin A/C (from S. Adam and R. Goldman).

### Southern blotting

The telomeric probe was made from the vector pHuR93 (61076, ATCC) containing 240 bp TTAGGG repeats. The 240-bp fragment was gel purified, and probe was labelled with radiolabelled dCTP using Ready-to-go labeling beads (GE Healthcare) according to the manufacturer.

Genomic DNA was prepared from 1 × 10^7^ patient cells, IMR90s or IMR90 cells expressing shEGFP(LT) or shLMNA(LT) by phenol/chloroform extraction and ethanol precipitation. DNA concentration was determined using multiple readings on a Nanodrop spectrophotometer and 3 μg of DNA extracted from each culture was digested overnight with 10 U RsaI and 10 U HinfI, leaving only telomeric DNA intact. Digested samples were run on a 0.7% agarose gel at 35 V overnight. Following electrophoresis, the gel was depurinated with 250 mM HCl and the DNA was denatured in 0.5 M NaOH/1.5 M NaCl for 25 min and 0.25 M NaOH/1.5 M NaCl for 25 min. The DNA fragments were blotted in a 0.25 M NaOH/1.5 M NaCl solution onto a nylon membrane for 12–15 h. After blotting, the single-stranded DNA fragments were crosslinked to the membrane by exposure to ultraviolet light and then soaked in 2 × SSC. The membrane was blocked by incubation in 10 ml preheated prehyb buffer (200 ml Starks buffer (5 × SSC, 25 mM NaPO_4_, pH 6.5, 5 × Denhardt’s solution, 0.25 mg ml^−1^ Torula RNA, 50% (v/v) formamide), 1% SDS, 0.5% milk) at 37 °C for 1 h rotating constantly. The membrane was then incubated at 45 °C overnight in hyb solution (80 ml prehyb buffer, 8% dextran sulphate) containing 25 ng of radiollabeled telomeric probe. Following incubation, the membrane was washed 15 min at 42 °C with 2 × SSC/0.1% SDS followed by another 15 min wash at room temperature. After washing the membrane with 0.1 × SSC/0.1% SDS for 10 min at 63 °C, the membrane was dried and the radioactively labelled telomeric sequence was visualized by autoradiography or using a phosphorimager (5–15 h).

### Fluorescence *in situ* hybridization

Probes for telomere FISH were made by nick translating pSXneo270(T2AG3) (Addgene plasmid 12403). Other probes used for FISH analysis were made from BAC or P1-derived artificial chromosomes (PACs) and are described in Supplementary Table 1. All probes were labelled by nick translation with DIG-11-dUTP (11558706910, Roche), BIO-16-dUTP (11093070910, Roche) or DNP-11-dUTP (NEL551001EA, PerkinElmer) with a Nick Translation Mix (11745808910, Roche) according to the manufacturer.

Mitotic chromosome FISH cultures were enriched for mitotic cells by Colcemid treatment (3 h at 0.1 μg ml^−1^). To prepare cells for mitotic FISH analysis, cells were trypsinized and resuspended in prewarmed hypotonic solution (75 mM KCl) for 5 min at 37 °C. Cells were pelleted and resuspended in fixative (1 part glacial acetic acid:3 parts absolute methanol). Cells were washed four additional times with fixative, and then resuspended in a volume of fixative about 10 times the size of the cell pellet. Cells were dropped onto a clean slide placed above a steam bath from a distance that was optimized for chromosome spreading (in our hands, this is roughly 10"). Fixative was allowed to evaporate, and the slide was removed from the steam bath and dried at room temperature overnight. For probe hybridization, slides were incubated with 100 μg ml^−1^ RNaseA in 2 × SSC at 40 °C for 1 h. Slides were then washed four times with 2 × SSC for 2 min each, and dehydrated using an ethanol series (70% EtOH, 80% EtOH, 95% EtOH) for 2 min each and allowed to air dry. Cells were denatured in 70% formamide/30% 4 × SSC for 2 min at 75 °C, and dehydration was repeated as before. Nick-translated probes (0.1 μg of each probe) were ethanol precipitated with 5 μg salmon sperm DNA and 1 μg human Cot1 DNA and resuspended in 10 μl of hybridization buffer (50% formamide/50%, 20% dextran sulfate in 4 × SSC) for 1 h at 37 °C. Probes were denatured at 75 °C for 5 min and then applied to denatured, air-dried slides. Slides were sealed with rubber cement and hybridization took place at 37 °C overnight. The next day, slides were washed with 50% formamide/50% 4 × SSC three times and 4 × SSC three times all at 40 °C. Cells were blocked with 2% bovine serum albumin (BSA) in 4 × SSC at 37 °C for 30 min, and then detected with the appropriate secondary antibodies diluted in block solution for 1 h at 37 °C. Slides were washed three times, for 5 min each with 4 × SSC/0.1% Triton X-100, and stained with 4',6-diamidino-2-phenylindole (DAPI) before mounting with Prolong Gold antifade reagent (Invitrogen). For a more detailed mitotic FISH protocol, see Espinosa and Le Beau^58^. Images in Fig. 1b were acquired on an N-SIM Structured Illumination Super-resolution Microscope (Nikon) at the Northwestern University Cell Imaging Facility.

Three-dimensional (3D) interphase FISH was performed based on the protocol of Cremer *et al.*^59^ In brief, cells grown on coverslips were fixed with 4% formaldehyde for 10 min and permeabilized with 0.5% Triton X-100 in PBS for 15 min at room temperature. Coverslips were then incubated in 20% glycerol in PBS for 4–5 h. Cells were subjected to four freeze–thaw cycles with liquid nitrogen, washed four times with PBS, treated with 0.1 N HCl for 10 min at room temperature, washed two times for 3 min with 2 × SSC and incubated overnight in 50% formamide/50% 4 × SSC at room temperature. Nick-translated probes were precipitated and resuspended in hybridization buffer as described for mitotic chromosome FISH analysis above. Probes were applied to cells, sealed with rubber cement and cells and probes were denatured together for 3 min at 75 °C. Hybridization occurred overnight at 37 °C, and the next day coverslips were removed and washed three times for 5 min with 2 × SSC at 37 °C and three times for 5 min with 0.1 × SSC at 60 °C. Cells were blocked with 4% BSA in 0.2% Tween 20/4 × SSC for 20 min at 37 °C, and detection was performed with the appropriate secondary antibodies in 1% BSA in 0.2% Tween 20/4 × SSC for 1 h at 37 °C. After washing in 0.2% Tween 20/4 × SSC, coverslips were stained with DAPI and mounted in Prolong Gold antifade reagent (Invitrogen). Images for 3D interphase FISH were acquired on an A1R Resonant Scanning Multispectral Confocal Microscope (Nikon) at the Northwestern University Cell Imaging Facility. Linear regression analysis was performed to identify the H17 probe as an outlier from the otherwise distance dependent co-localization frequency. Nuclear volumes were calculated using the ImageJ^60^ Object Counter 3D plugin^61^ ( http://imagejdocu.tudor.lu/doku.php?id=plugin:analysis:3d_object_counter:start).

Secondary antibodies used for detection: 1:250 sheep α-DIG fluorescein (11207741910, Roche), 1:250 mouse α-Bio Alexa Fluor 647 (200-602-211, Jackson ImmunoResearch), 1:1,000 rabbit α-DNP-KLH (A-6430, Molecular Probes) and 1:1,000 goat α-rabbit Alexa Fluor 594 (A-11012, Molecular Probes).

For inverted chromosome-end analysis, a chromosome was considered in the ‘linear’ orientation if the two FISH signals co-localized or showed the orientation predicted by the linear genome sequence. Only chromosomes where the FISH signal that was predicted to be closer to the telomere based on the linear genome sequence was actually observed closer to the centromere were considered ‘inverted’. Confidence intervals were calculated using the Clopper–Pearson method. Groups were compared for statistical significance using a one-tailed, unpaired *t*-test.

### Telomere pull-down assay

The telomere pull-down assay was adapted from Déjardin and Kingston^26^. A 15-cm cell culture dish of 80% confluent cells were used per pull down. Briefly, cells were crosslinked for 10 min at room temperature in 1% formaldehyde and repeatedly washed in PBS with protease inhibitors (Roche Complete Protease Inhibitor Cocktail). Cells were resuspended in sucrose solution (0.3 M sucrose/10 mM HEPES-NaOH, pH 7.9/1% Triton X-100/2 mM magnesium acetate), homogenized with a tight pestle (B) 20 times and pelleted and resuspended in glycerol buffer (25% glycerol/10 mM HEPES-NaOH, pH 7.9/1% Triton X-100/2 mM magnesium acetate). The pellet was then treated with 1.5 mg ml^−1^ RNaseA in 0.5% Triton X-100/PBS for 2 h at room temperature. The pellet was washed multiple times in PBS with protease inhibitors and washed and resuspended in high-salt lysis buffer (10 mM HEPES-NaOH, pH 7.9/100 mM NaCl/2 mM EDTA/1 mM EGTA/0.2% SDS/0.1% sodium sarkosyl/protease inhibitors). At this point, samples were sonicated to obtain 200–500 bp fragments (Branson Sonifier 450 with 8 and 15 s sonications, output 2). After pre-clearing chromatin with High Capacity Streptavidin Agarose Resin (Thermo Scientific) and filtering with Sephacryl S-400 High Resolution medium (GE Healthcare), an input sample was removed and 2 μl of a 100 μM LNA telomere or scramble probe (Exiqon custom probes synthesized as described^26^) was hybridized to the remaining sample. After pull down with Dynabeads MyONE Streptavidin C1 (Invitrogen), DNA was eluted with 95% formamide/10 mM EDTA for 10 min at 90 °C. Sample was diluted to a final concentration of 50 mM Tris, pH 7.5, 0.5% SDS and 1 mg ml^−1^ proteinase K and incubated at 4 h at 65 °C. Sample was purified by phenol:chloroform extraction and ethanol precipitation and DNA was resuspended in 10 mM Tris, pH 8.0 and analysed by qPCR. Reported data represent the mean±s.d. of three biological replicates.

Precipitated DNA was quantified by qPCR analysis using a Roche LightCycler 480 Real-Time PCR System and SYBR Green I Master mix (04707515001, Roche). The amount of DNA was quantified relative to input signal, and all qPCR reactions were performed in triplicate. Primers used for qPCR analysis are included in Supplementary Table 2.

### Comparative genome hybridization

CGH was performed as previously described^62^, using genomic DNA isolated from wild-type IMR90 cells as reference and shEGFP(LT) and shLMNA(LT) as donors. The BAC array used has been described^62^, and contains a total of 4,153 different BAC clones with a median spacing of 413 kb and a mean spacing of 677 kb.

### ChIP and qPCR analysis

IMR90s (2 × 10^6^ cells per ChIP) were trypsinized, washed with PBS and fixed for 10 min at room temperature in 1% formaldehyde. Fixation was quenched with glycine and after washing the cells with cold PBS, cells were resuspended in lysis buffer (50 mM Tris HCl, pH 8.0, 10 mM EDTA, 1% SDS, Complete Protease Inhibitor Cocktail (Roche)) for 25 min on ice. Lysates were sonicated to generate 200–1,000 bp DNA fragments, and then diluted sixfold in IP dilution buffer (0.01% SDS, 1.1% Triton X-100, 1.2 mM EDTA, 16.7 mM Tris HCl, pH 8, 167 mM NaCl). Diluted lysate was precleared with protein G Dynabeads (Invitrogen) for 2 h at 4 °C. At this point, an input sample was removed. The remaining lysate was incubated overnight with antibody at 4 °C. Protein G Dynabeads were added for 2 h at 4 °C. Beads were washed three times with low-salt wash buffer (0.1% SDS, 1% Triton X-100, 2 mM EDTA, 20 mM Tris HCl, pH 8, 150 mM NaCl, protease inhibitor cocktail), two times with high-salt wash buffer (0.1% SDS, 1% Triton X-100, 2 mM EDTA, 20 mM Tris HCl, pH 8, 500 mM NaCl, protease inhibitor cocktail), two times with LiCl wash buffer (10 mM Tris HCl, pH 8, 1 mM EDTA, 0.25 M LiCl, 1% NP40, 1% deoxycholic acid, protease inhibitor cocktail) and one time with TE (10mM Tris HCl, 1mM EDTA). Precipitated material was eluted from beads twice with 0.1 M NaHCO_3_/1% SDS at 37 °C for 10 min. Crosslinking was reversed at 65 °C overnight, material was digested with proteinase K and DNA was purified by phenol–chloroform extraction and ethanol precipitation. Three biological iterations were performed for each experiment and the values reported represent the mean of these replicates±s.e.m. Antibodies: polyclonal rabbit α-TRF2 (NB110-57130, Novus Biologicals), monoclonal mouse α-lamin A/C (from R. Goldman and S. Adam), normal rabbit IgG (sc-2027, Santa Cruz) and normal mouse IgG (sc-2025, Santa Cruz).

Precipitated DNA was quantified by qPCR analysis using a Roche LightCycler 480 Real-Time PCR System and SYBR Green I Master mix (04707515001, Roche). ChIP samples were quantified relative to input signal, and all reactions were performed in triplicate. Statistical significance was determined using a one-tailed, paired *t*-test. Primers used for qPCR analysis are included in Supplementary Table 2.

## Additional information

**How to cite this article:** Wood, A. M. *et al.* TRF2 and lamin A/C interact to facilitate the functional organization of chromosome ends. *Nat. Commun.* 5:5467 doi: 10.1038/ncomms6467 (2014).

## Supplementary Material

Supplementary Information Supplementary Figures 1-8, Supplementary Tables 1-2, and Supplementary References
