# Supplementary material for: TRF2 and lamin A/C interact to facilitate the functional organization of chromosome ends
Source: Nat Commun. 2014 Nov 17;5:5467. doi: 10.1038/ncomms6467 (PMC4235626; doi:10.1038/ncomms6467)
Supplement: Supplementary Information — Supplementary Figures 1-8, Supplementary Tables 1-2, and Supplementary References [file ncomms6467-s1.pdf]

Supplementary Figures

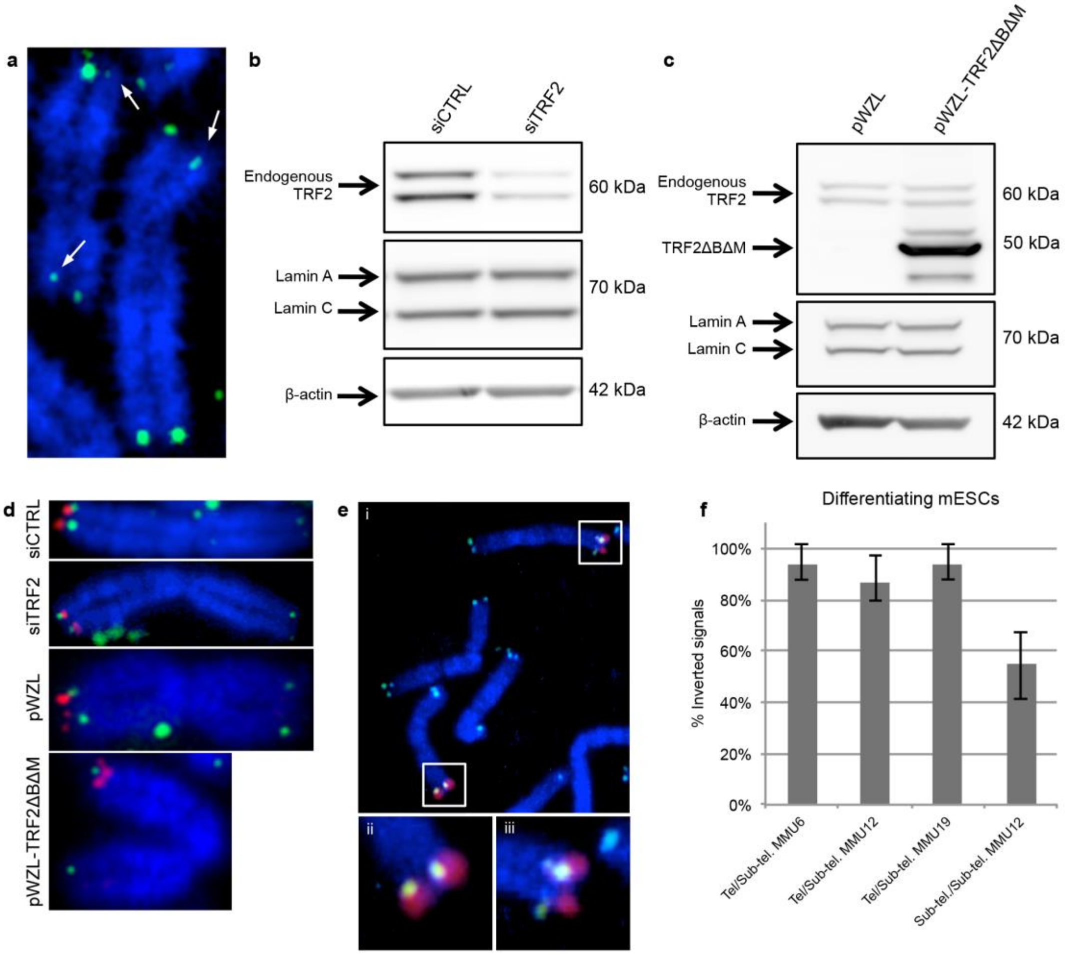

Supplementary Figure 1. Chromosome inversion at mitotic chromosome ends. a, Mitotic chromosomes with telomere FISH probe (green) depicting the variance in telomere signal positioning in relation to the chromosome end. Arrows represent ends where the telomere signal is far from the chromosome end. DNA is stained with DAPI. **b,** Western blot depicting TRF2 knockdown after treating IMR90s for 72 hrs with siTRF2 or a control siRNA (siCTRL). At this time point lamin A/C levels are not affected. β-actin is used as a loading control. **c,** Western blot showing TRF2ΔBΔM overexpression in IMR90s 5 days after selection. Endogenous TRF2 and lamin A/C levels are not affected. β-actin is used as a loading control. For **d** and **e**, FISH analysis was performed on mitotic chromosomes with a telomere FISH probe (green) and a subtelomeric FISH probe (red). DNA is stained with DAPI (blue). **d,** Mitotic HSA1 from IMR90s showing an inverted chromosome-end structure after siCTRL or pWZL treatment and a linear chromosome-end structure after siTRF2 or pWZL-TRF2ΔBΔM treatment. **e,** Mitotic chromosome FISH analysis in activated lymphocytes with the chromosome end of the two HSA14 alleles enlarged in images on the bottom (ii and iii). **f,** Mitotic chromosome FISH analysis from mouse embryonic stem cells (mESCs) allowed to spontaneously differentiate. Error bars represent 95% confidence intervals.

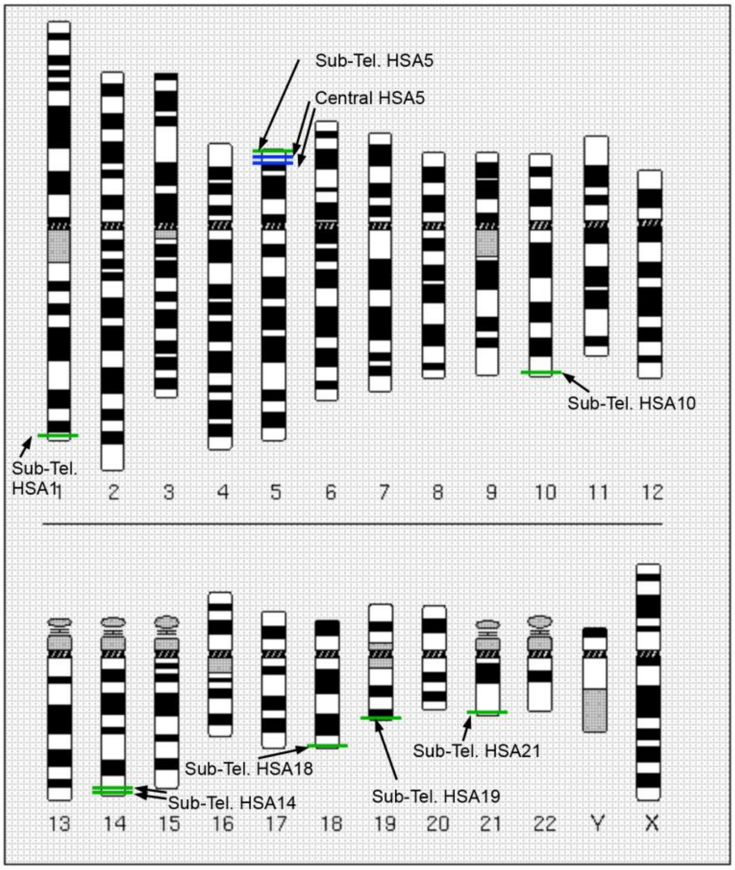

Supplementary Figure 2. BAC Positioning for FISH probes. Location of BACs used for mitotic chromosome FISH analysis. Ideogram from http://www.pathology.washington.edu/research/cytopages/idiograms/human/ copyright © 1994 David Adler.

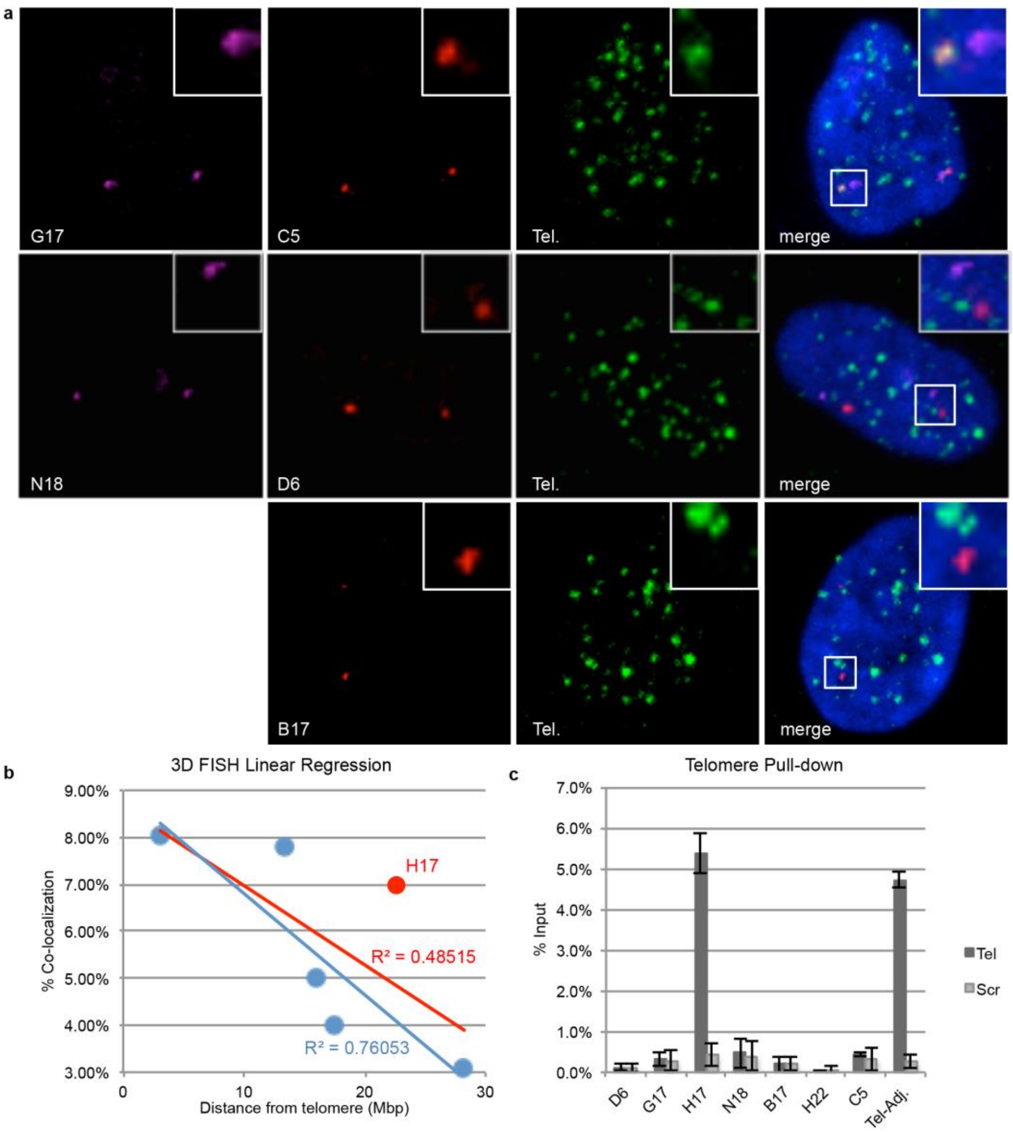

Supplementary Figure 3. Chromosome-end organisation in interphase nuclei. a, Representative images of IMR90 3D FISH analysis for HSA1 BACs (G17, C5, N18, D6 and B17). One allele (white box) for each image is enlarged in the upper right hand corner depicting C5 co-localisation with the telomere signal, but not the other BACs. **b,** Linear regression analysis of 3D interphase FISH data depicted in Fig. 2b. The co-localisation frequency between each probe and the telomere is graphed in relation to its genomic distance from the telomere. Since the D6 probe is beyond the distance dependence for co-localisation it was excluded from this analysis. The H17 probe is labeled and shown in red. Linear regression analysis of all 6 points gave a R^2 value of 0.48515 (red line) and in this analysis, H17 was the only probe >1.5 standard deviations from the regression line. Removal of the H17 probe increased the goodness of fit of the linear regression with an R^2 value of 0.76053 (blue line). Therefore, H17 was deemed an outlier. **c,** Telomere pull-down assay depicting the crosslinking frequency of regions of the q arm of HSA1 and telomeric DNA. Pull-down was performed with a telomeric probe (dark gray) or a scrambled probe (light gray). The H17 region shows a substantial enrichment for pull-down over the other non-telomeric regions, but similar to that of a telomere adjacent region (Tel-Adj.) that contains a high density of telomeric repeats.

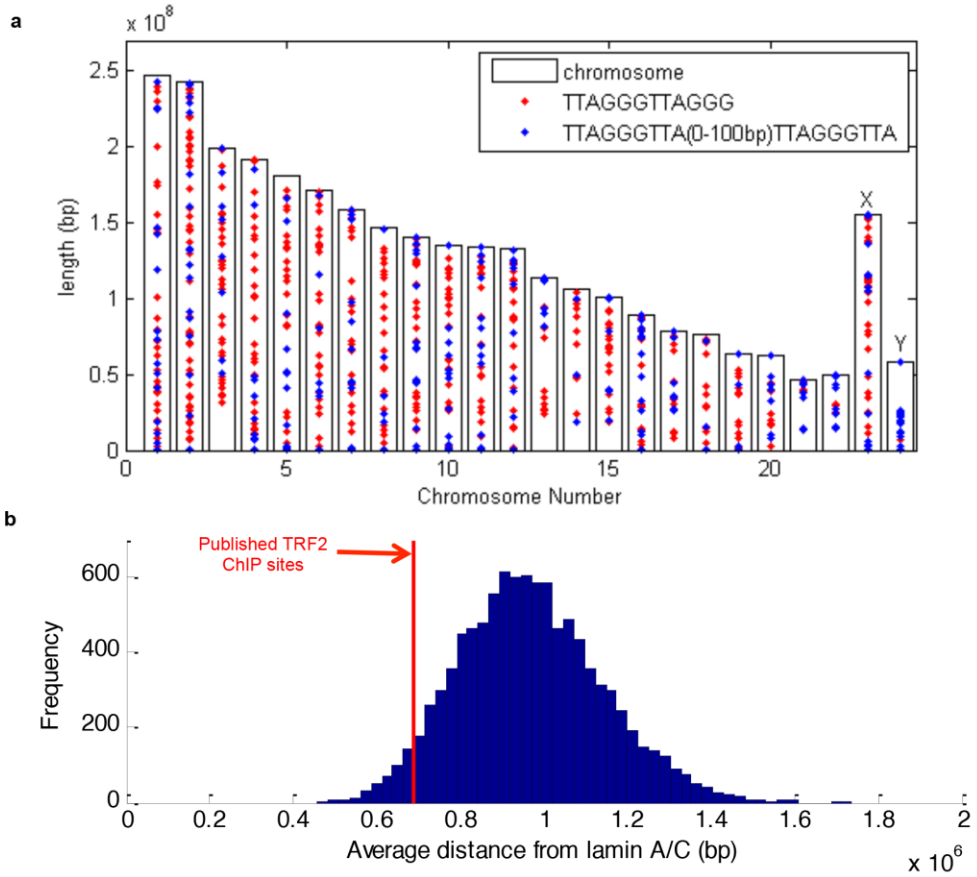

Supplementary Figure 4. Bioinformatic analysis of TRF2 and lamin A/C sites. a, Location of ITSs in the human genome. Total there are 2920 ITSs. **b,** The distance of the 112 published interstitial TRF2 binding sites[1,2] to lamin A/C sites[3]. The position of each site was randomised within its corresponding chromosome and the distance to the nearest lamin A/C site was calculated. The randomisation was repeated 10,000 times and the average distance for each randomised data set was plotted as a histogram (blue). The average distance between the actual TRF2 ChIP sites and lamin A/C sites was also calculated and is represented by a red bar. The actual TRF2 ChIP sites are closer to lamin A/C domains than expected based on the randomised data sets ($p=0.043$).

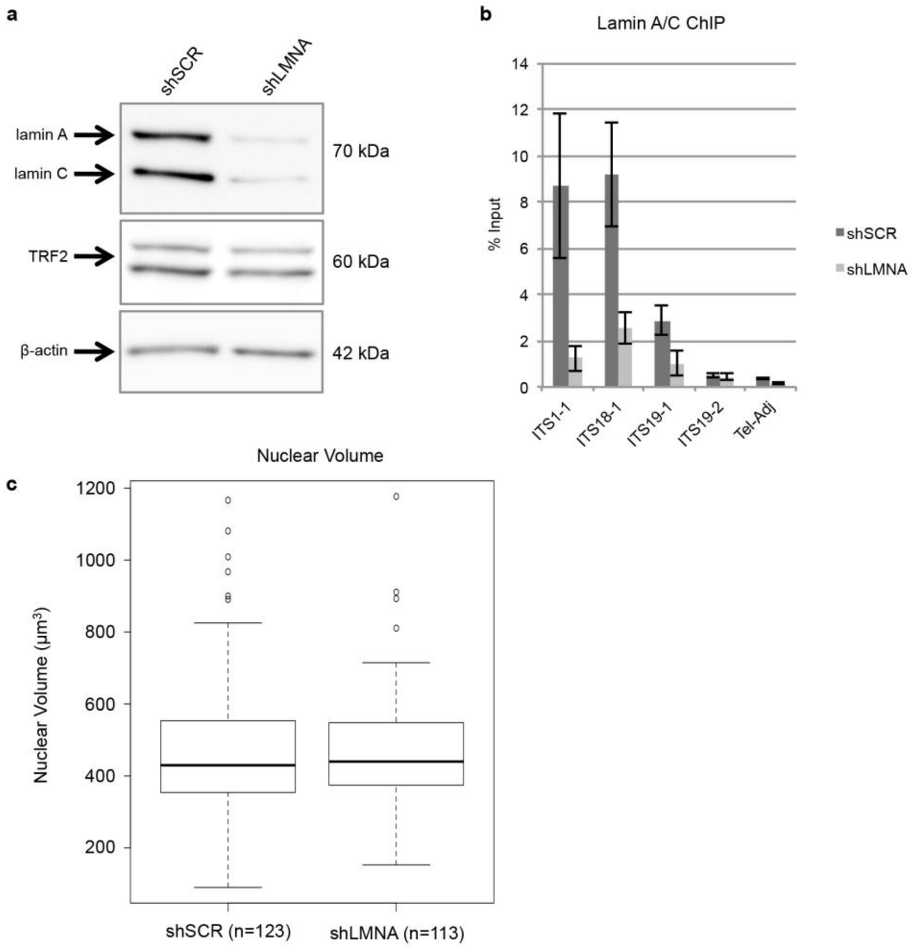

Supplementary Figure 5. Lamin A/C knockdown and ChIP analysis at ITSs. a, shLMNA knockdown in IMR90s for 6 days after selection results in a reduction in lamin A/C protein levels, but not TRF2. β-actin is used as a loading control. **b,** shLMNA knockdown leads to a decrease in lamin A/C association with ITSs. Results are mean ± s.e.m. (n=3). **c,** Nuclear volume was measured using the ImageJ Object Counter 3D plugin for nuclei treated with shSCR or shLMNA and harvested 6 days after selection. At this time point, no difference was seen in nuclear volume.

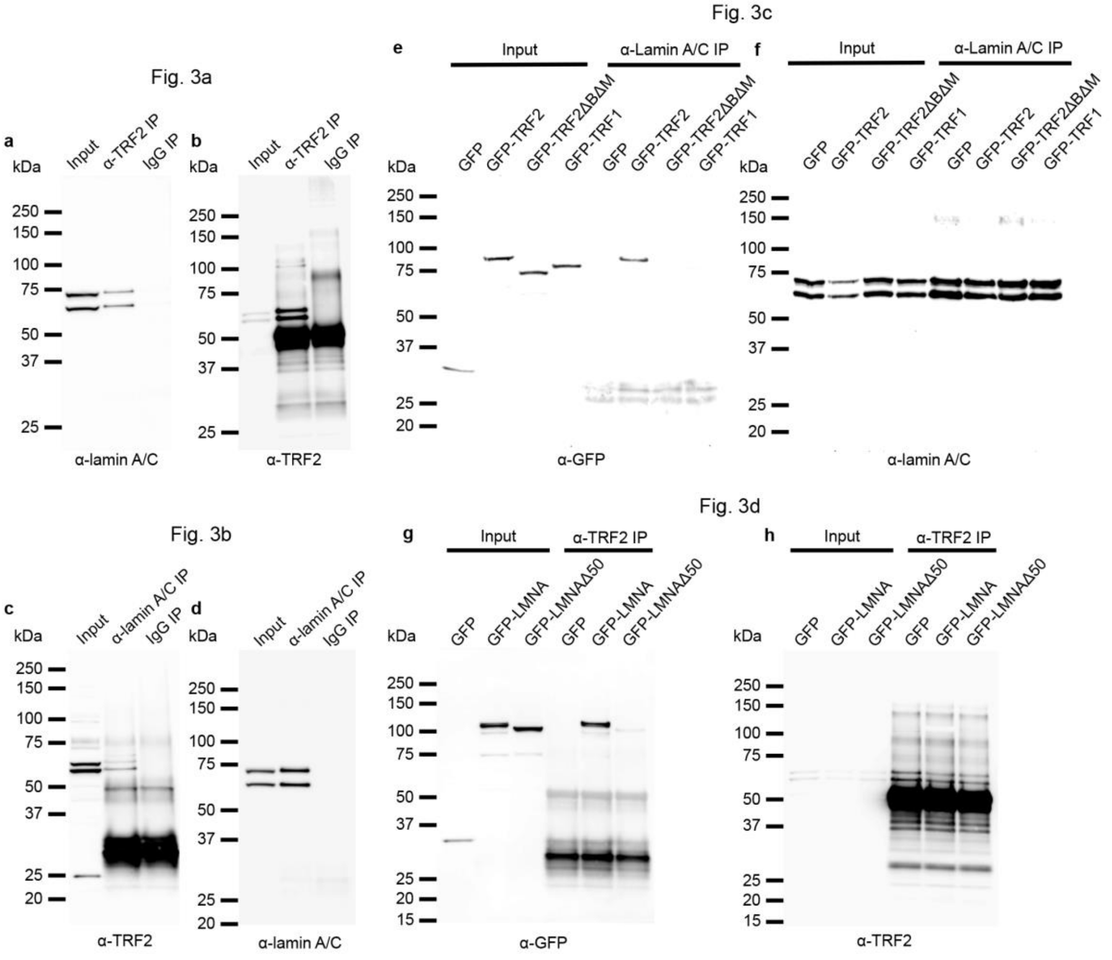

Supplementary Figure 6. Western blots from Fig. 3. Full blots with molecular weight markers are shown for each of the cropped westerns in Fig. 3. **a** and **b,** are Fig.3a. **c** and **d,** are Fig 3b. **e** and **f,** are Fig. 3c. **g** and **e,** are Fig. 3d.

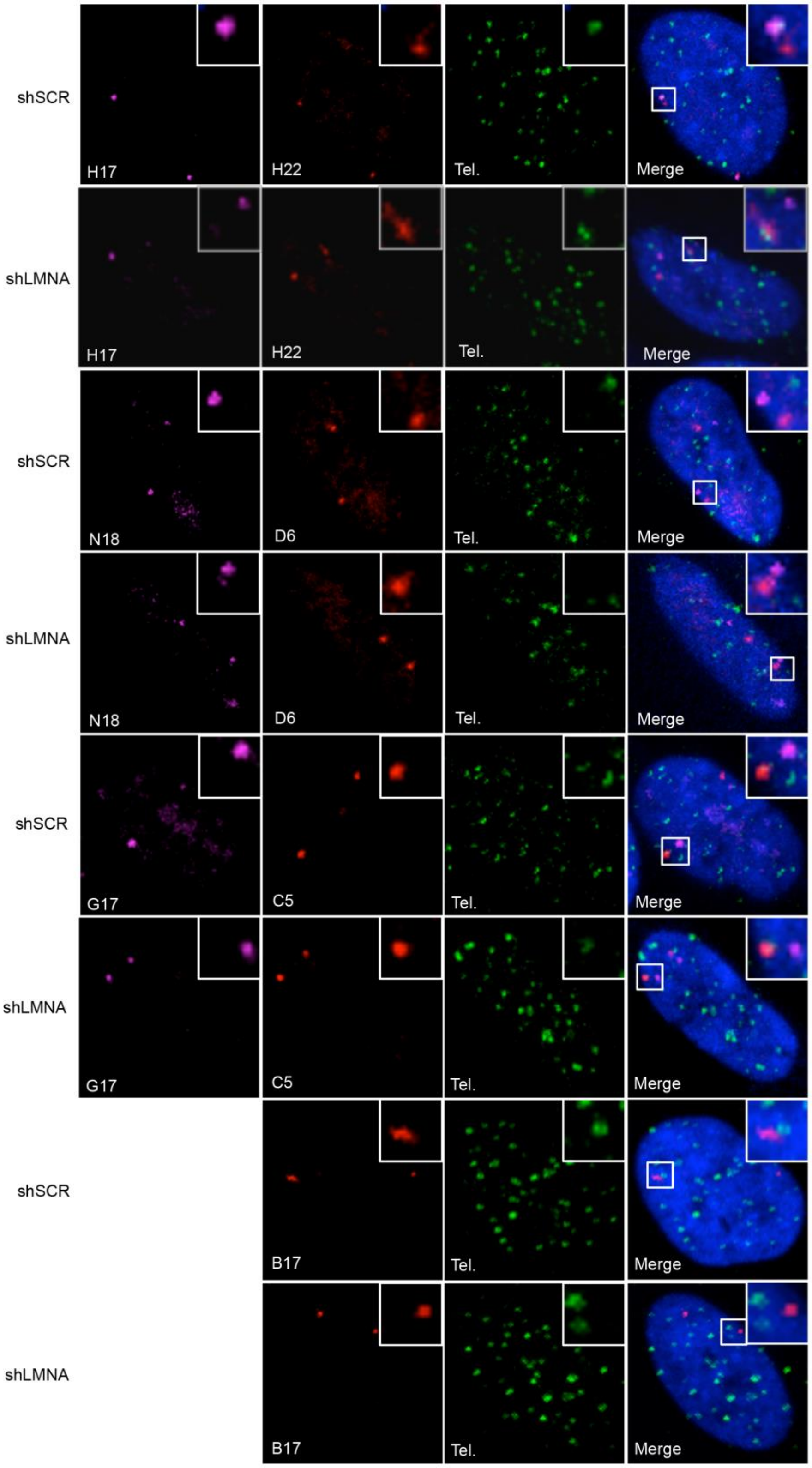

shSCR
H17
H22
Tel.
Merge
shLMNA
H17
H22
Tel.
Merge
shSCR
N18
D6
Tel.
Merge
shLMNA
N18
D6
Tel.
Merge
shSCR
G17
C5
Tel.
Merge
shLMNA
G17
C5
Tel.
Merge
shSCR
B17
Tel.
Merge
shLMNA
B17
Tel.
Merge

Supplementary Figure 7. Chromosome-end organisation in interphase nuclei after lamin A/C knockdown. 3D FISH analysis of IMR90s treated with either shLMNA or shSCR for 6 days after selection. BACs are shown in magenta and red as indicated and telomere FISH is in green. DNA is stained with DAPI. Representative images are shown for each condition with one allele (white box) enlarged in the upper right hand corner.

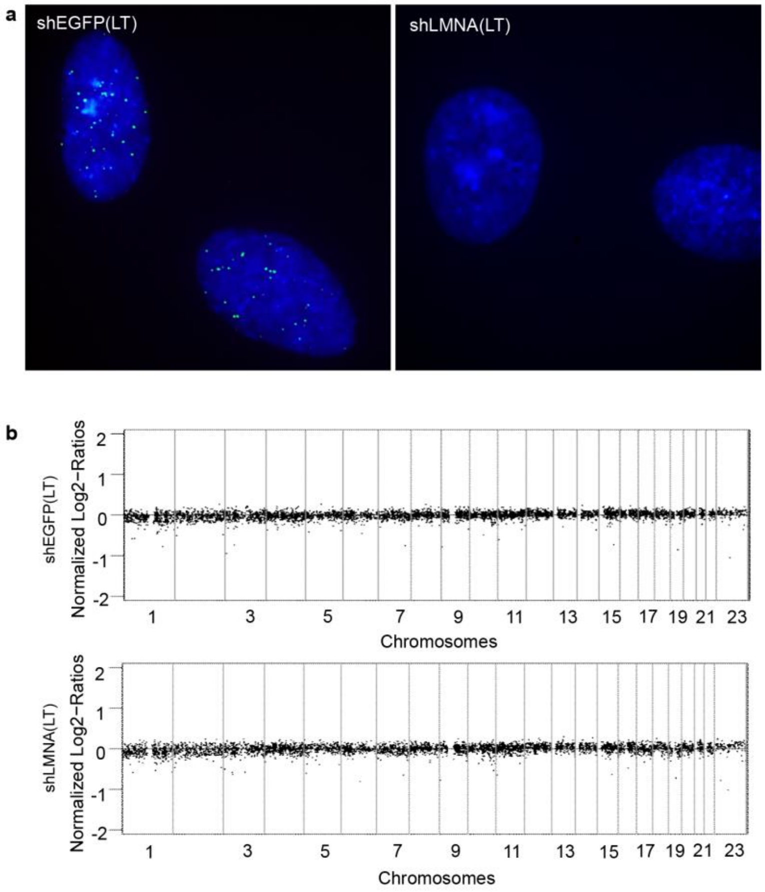

Supplementary Figure 8. Lamin A/C is necessary for telomere stability. a, Telomeric FISH analysis in shEGFP(LT) and shLMNA(LT) IMR90s. There is a reduction in the number of telomeric foci per nucleus in shLMNA(LT) cells. **b,** Comparative genome hybridisation analysis with wildtype IMR90 DNA as a standard for shEGFP(LT) and shLMNA(LT) IMR90 DNA. This analysis reveals no gross genomic instability.

Supplementary Tables

Supplementary Table 1. BAC/PACs used to generate FISH probes.

BAC/PAC	Genomic location	Description	Reference
RP11-91C5	1q44<br>HSA1:246,183,705-246,342,751	Subtelomere HSA1	CHORI BAC/PAC Resources Center
RP11-656H22	1q42<br>HSA1:235,843,027-236,012,944	q arm HSA1	CHORI BAC/PAC Resources Center
RP11-109B17	1q42<br>HSA1:233,346,567-233,540,973	q arm HSA1	CHORI BAC/PAC Resources Center
RP11-88N18	1q42<br>HSA1:231,727,974-231,884,507	q arm HSA1	CHORI BAC/PAC Resources Center
RP11-31H17	1q42<br>HSA1:226,570,902-226,750,697	q arm HSA1	CHORI BAC/PAC Resources Center
RP11-780G17	1q41<br>HSA1:221,023,302-221,204,375	q arm HSA1	CHORI BAC/PAC Resources Center
RP11-196D6	1q32<br>HSA1:202,999,169-203,159,216	q arm HSA1	CHORI BAC/PAC Resources Center
RP11-91C19	18q23<br>HSA18: 76,920,278-77,080,078	Subtelomere HSA18	CHORI BAC/PAC Resources Center
RP11-91H11	19q13.4<br>HSA19: 58,531,428-58,696,029	Subtelomere HSA19	CHORI BAC/PAC Resources Center
P24H17	5p15	Subtelomere HSA5	
B2039G12	5p15	Central HSA5	
B2247C11	5p15	Central HSA5	
B261B16	10q26	Subtelomere HSA10	
B200D12	14q32	Subtelomere HSA14	Gozzetti et al. 2002[4]
B158A2	14q32	Subtelomere HSA14	Gozzetti et al. 2002[4]
P2H4	21q22	Subtelomere HSA21	Gozzetti et al. 2002[4]

Supplementary Table 2. Primers for ChIP-qPCR and telomere-pull down analyses.

Region	Forward Primer (5' to 3')	Reverse Primer (5' to 3')
H17/ITS1-1	GCCCCTGGGCCTGTAAAGATAACA	AGGATCCTCGCCTTGGCACA
ITS18-1	TCCATGTGGTCTCTCTGTCTGGACT	GACGGGCCAGACTGTTGCAT
ITS19-1	GAACAAGTGCGCGTTTCAGT	ACTGTCGGCTCTCCCATTTG
ITS19-2	GTCACGTGCACACAGGAAAC	ACCCAGAAAACTCAGCCTCG
Tel-Adj[5]	CCCCTTGCCTTGGGAGAA	GAAAGCAAAAGCCCCTCTGA
H22/HSA1-2	TGTGAGCCTGAGGAACTGCAAGC	AGTGCAAGCTGGTCGAACAGGAA
RPLP0	GCGCCCATCTAACTAGCACA	TCGCGACCCTACTTAAAGGC
C5	GGTTCGGGTCTTGTCGAACT	CCCCCATTTCCAGTCTGTCC
B17	GGGCTCCATGATTCCCAACA	AGAACATGGCCAGGACACAG
N18	ATTACCAGGGCAAGCAGGTC	GCTGCTCGAGGACTCTTGTT
G17	TCTGGGGTTGGTTCATGTCG	GAGGGGGACTTGGGTAGGAT
D6	CCGGAAAGAATGGGACTGCT	TGCTTGGGAACCCCAAAAGT

References:

1 Simonet, T. *et al.* The human TTAGGG repeat factors 1 and 2 bind to a subset of interstitial telomeric sequences and satellite repeats. *Cell research* **21**, 1028-1038, doi:10.1038/cr.2011.40 (2011).

2 Yang, D. *et al.* Human telomeric proteins occupy selective interstitial sites. *Cell research* **21**, 1013-1027, doi:10.1038/cr.2011.39 (2011).

3 McCord, R. *et al.* Correlated alterations in genome organization, histone methylation, and DNA-lamin A/C interactions in Hutchinson-Gilford progeria syndrome. *Genome research* **23**, 260-269, doi:10.1101/gr.138032.112 (2013).

4 Gozzetti, A. *et al.* Identification of novel cryptic translocations involving IGH in B-cell non-Hodgkin's lymphomas. *Cancer Res* **62**, 5523-5527 (2002).

5 Deng, W. *et al.* Controlling long-range genomic interactions at a native locus by targeted tethering of a looping factor. *Cell* **149**, 1233-1244, doi:10.1016/j.cell.2012.03.051 (2012).
